# Click chemistry compared to thiol chemistry for the synthesis of site-selective glycoconjugate vaccines using CRM_197_ as carrier protein

**DOI:** 10.1007/s10719-020-09930-2

**Published:** 2020-06-13

**Authors:** G. Stefanetti, M. Allan, A. Usera, F. Micoli

**Affiliations:** 1 Department of Immunology, Blavatnik Institute, Harvard Medical School, Boston, MA 02115 USA; 2 Novartis Institutes for BioMedical Research, 250 Massachusetts Avenue, Cambridge, MA 02139 USA; 3 GSK Vaccines Institute For Global Health (GVGH) S.r.l, Via Fiorentina 1, Siena, 53100 Italy

**Keywords:** Glycoconjugate vaccine, Click chemistry, Thiol chemistry, Conjugation chemistry, *Salmonella* Typhimurium, O-antigen, CRM_197_

## Abstract

Conjugation chemistry is one of the main parameters affecting immunogenicity of glycoconjugate vaccines and a rational approach toward a deeper understanding of their mechanism of action will greatly benefit from highly-defined and well-characterized structures. Herein, different conjugation methods were investigated with the aim of controlling glycosylation site and glycosylation density on the carrier protein. *S.* Typhimurium lipopolysaccharide O-Antigen and CRM_197_ carrier protein were used as models. In particular, thiol and click chemistry were examined, both involving the linkage of the terminal reducing sugar unit of the O-Antigen chain to different amino acids on the carrier protein. Thiol chemistry allowed O-Antigen conjugation only when the carrier protein was activated on the lysines and with a relative high number of linkers, while click chemistry allowed conjugate generation even when just one position on the protein was activated and to both lysine and tyrosine sites. The study highlights click chemistry as a leading approach for the synthesis of well-defined glycoconjugates, useful to investigate the relationship between conjugate design and immune response.

## Introduction

Glycoconjugate vaccines are important therapeutics for the prevention of infectious disease from severe pathogens like *Neisseria meningitidis*, *Haemophilus influenza* and *Streptococcus pneumoniae* [1, 2]. They require the covalent linking of a sugar antigen to a carrier protein, which can be obtained by different strategies influencing both the efficiency of conjugation and the structure of the glycoconjugate, with an important impact on immunogenicity [3]. Traditional glycoconjugate approaches involve random linkage of the sugar hapten to a carrier protein, or end-group modification of the saccharide chain to achieve better control and characterization of the resulting vaccine. More defined constructs where the covalent linking between sugar and the protein is limited to well-established attachment sites are highly desirable. They allow a better characterization of the product by physicochemical techniques facilitating the control of manufacturing consistency. Furthermore, the role of glycosylation site on the protein still has to be fully understood and such well-defined products can support studies to further investigate the relationship between glycoconjugate design and immune response. Hence, increasing attention has been made to control the conjugation site [4–6] and provide structurally-defined products not only in their saccharide component but also in the attachment point to the protein [6–9].

We have recently synthesized well-defined O-antigen (OAg)-based glycoconjugate vaccines to protect against nontyphoidal *Salmonella* (NTS) serovar Typhimurium, with CRM_197_ as carrier protein [6]. NTS is the commonest cause of invasive bacteremia in Africa [10, 11], particularly affecting young children and HIV-infected adults, and OAg-based glycoconjugates represent a leading approach for the development of a vaccine against NTS [12–15].

Importantly, we found that site-selective single or double attachment of OAg to CRM_197_ was enough to generate levels of functional antibodies in mice, with titers comparable to the IgG induced by more complex random conjugates, and that the attachment site on the carrier protein plays a role on the immunogenicity.

In this study we compared four different orthogonal conjugation chemistries for the generation of selective OAg-based glycoconjugate vaccines against NTS, with the aim to identify efficient strategies to link a different number of sugar chains to defined amino acid sites on the protein. In all the constructs, *S.* Typhimurium OAg was end-terminally conjugated to CRM_197_ as carrier protein via the KDO (3-deoxy-D-manno-octulosonic acid) unit. CRM_197_ is a 58 kDa nontoxic mutant of diphtheria toxin and was selected as carrier protein for this investigation because of its defined structure and extensive use for licensed glycoconjugate vaccines and other vaccines in development [16, 17].

Two methods used refer to thiol chemistry, the thiol-maleimide addition and thioalkylation with halides, while two methods were based on the Huisgen 1,3-dipolar cycloaddition, which was investigated both copper-catalyzed and by the strain promoted variant. Click chemistry has been used for the synthesis of glycoconjugate vaccines only in recent times [6, 8, 9, 18], while the generation of a stable thioether bond by thiol-maleimide addition [19] or thioalkylation with halides [20, 21] are more common conjugation strategies which have been also used for the development of licensed glycoconjugate vaccines against *Haemophilus influenza* type b [19, 20].

Differently from thiol chemistry, strain promoted copper-free click chemistry resulted in an efficient coupling strategy both in regulating the glycosylation density on the final conjugate and in selectively targeting determined amino acid sites on the carrier protein.

## Materials and methods

### Reagents

The following chemicals were used in this study: cystamine dihydrochloride, 1-ethyl-3-(3-dimethylaminopropyl)carbodiimide (EDAC), (+)-sodium L-ascorbate, 2-(N-morpholino)ethanesulfonic acid (MES), N-hydroxysuccinimide (NHS), tris(2-carboxyethyl)phosphine hydrochloride solution (TCEP), propargylamine, copper(II) sulfate pentahydrate (CuSO_4_·5H_2_O), tris(3-hydroxypropyltriazolylmethyl)amine (THPTA), phosphate buffer solution (PBS), adipic acid dihydrazide (ADH), sodium cyanoborohydride (NaBH_3_CN), dimethyl sulfoxide (DMSO), sodium phosphate monobasic (NaH_2_PO_4_) [Sigma]; triplex III (EDTA) [Merck]; N-[β-maleimidopropionic acid] hydrazide trifluoroacetic acid salt (BMPH) [Thermo]; N-[ε-maleimidocaproyloxy]succinimide ester (EMCS), succinimidyl 3-(bromoacetamido)propionate (SBAP), NHS-PEG4-N_3_ [Pierce], Click-easyTM BCN N-hydroxysuccinimide ester I (BCN NHS I) [Berry & Associates]; DL-1,4-Dithiothreitol (DTT) [Invitrogen]. CRM_197_ was obtained from Novartis Vaccines and Diagnostics (NV&D).

### OAg purification and characterization

*S*. Typhimurium OAg was purified as previously described [22], following fermentation of the animal-derived isolate 2192, obtained from the University of Calgary. OAg resulted pure from proteins (< 1% w/w by micro BCA), nucleic acids (< 1% w/w by A_260_) and endotoxins (< 0.1 UI/µg by LAL). 2192 OAg was fully characterized [23]: it showed an average molecular weight of 20.5 kDa based on HPLC-SEC analysis dRI profile with dextrans as standard, was 100% O-acetylated on C-2 abequose and 24% glucosylated. Amino groups were detected by TNBS colorimetric method [24, 25] probably as pyrophosphoethanolamine residues in the core region, with a molar ratio of 0.38 respect to N-acetyl glucosamine, unique sugar of the core [23].

### Synthesis and characterization of derivatized CRM_197_

#### CRM_197_-BMPH via EDAC chemistry

CRM_197_ was solubilized in MES 500 mM pH 6.0 (12 mg/mL); BMPH (42 mg/mL, molar ratio BMPH/COOH groups CRM_197_ = 10.42) and EDAC (3 mg/mL, molar ratio EDAC/COOH groups CRM_197_ = 1.15) were added. Mixture was stirred for 1 h at RT, and then purified by desalting against NaH_2_PO_4_ 100 mM EDTA 10 mM pH 7.0 on a HiPrep 26/10 desalting column 53 mL, prepacked with Sephadex G-25 Superfine (G-25 53 mL) [GE Healthcare].

#### CRM_197_-BMPH via EDAC/NHS chemistry

CRM_197_ was solubilized in MES 600 mM pH 6.0 (15.56 mg/mL); NHS (10.8 mg/mL, molar ratio NHS/COOH groups CRM_197_ = 5.36) and EDAC (6.2 mg/mL, molar ratio EDAC/COOH groups CRM_197_ = 1.83) were added and the solution mixed at RT for 1 h. After this time, BMPH (2.8 mg/mL, molar ratio BMPH/COOH groups CRM_197_ = 0.53) was added and the solution stirred for 2 h at RT. The mixture was purified by desalting on a PD-10 desalting column (PD 10) [GE Healthcare] against NaH_2_PO_4_ 100 mM EDTA 1 mM pH 7.0.

#### CRM_197_-EMCS

CRM_197_ was solubilized in NaH_2_PO_4_ 100 mM EDTA 1 mM pH 8.0 (4.7 mg/mL); EMCS was added (0.19 mg/mL, molar ratio EMCS/Lysine groups on CRM_197_ = 0.2) after being solubilized in DMSO (final DMSO concentration of 6% v/v). Mixture was stirred for 2 h at RT, and then purified by desalting (G-25 53 mL column) against NaH_2_PO_4_ 100 mM EDTA 1 mM pH 7.0.

#### CRM_197_-SBAP

CRM_197_ was solubilized in NaH_2_PO_4_ 100 mM EDTA 1 mM pH 8.0 (4.7 mg/mL); SBAP was added (0.3 mg/mL, molar ratio SBAP/Lysine groups on CRM_197_ = 0.3) after being solubilized in DMSO (final DMSO concentration of 4% v/v). Mixture was stirred for 3 h at RT, and then purified by desalting (G-25 53 mL column) against NaH_2_PO_4_ 100 mM EDTA 1 mM pH 7.0.

#### CRM_197_-N3 by controlled insertion of linkers on Lys (+ 3.8, + 5.2, + 7.1 and + 10)

CMR_197_ was solubilized in NaH_2_PO_4_ 400 mM pH 7.2 (20 mg/mL), and NHS-PEG4-N_3_ was added (linker solubilized in DMSO with final DMSO concentration in the mixture of 1% v/v). Different amount of NHS-PEG4-N_3_ were used to achieve a different degree of derivation on the protein with a molar ratio NHS-PEG4-N_3_/Lysine groups CRM_197_ of 0.18, 0.26, 0.39, respectively. After mixing at RT for 8 h, the mixture was purified by desalting on a G25 53 mL column against NaH_2_PO_4_ 100 mM pH 7.2.

#### CRM_197_-maleimide

4-(4-(2,5-dioxo-2,5-dihydro-1H-pyrrol-1-yl)butyl)-3H-1,2,4-triazole-3,5(4H)-dione [26] (20 mM in CH_3_CN, freshly prepared) was added dropwise, sequentially every minute in 10 copies, to a solution of CRM_197_ at 4 °C (0.85 mg/mL in Tris HCl 500 mM pH 7.4), targeting tyrosine resiudes with a molar ratio to CRM_197_ of 5:1. The mixture was stirred at 4 °C for 15 min and then desalted on Zeba 7K MWCO spin column [Pierce] with PBS pH 7.4 as the eluting buffer for three times.

#### CRM_197_-N3 by selective insertion of linkers on Lys (+ 1)

CRM_197_-N_3_ was synthesized as previously reported [6]. In brief, the linker ZQG-NH-PEG3-N3 was first synthesized and characterized [6]. CRM_197_ (32 mg/mL, 32 µL) was then added to ZQG-NH-(PEG)3-N3 (2 mg/mL, 1 mL) in Tris 100 mM pH 8 and 100 µL of mTGase (stock of 50 mg/mL in PBS prepared from commercial 1% mTGase in maltocyclodextrin) were added. Reaction was incubated at 25° C for 18 h. The mixture was purified by size exclusion chromatography (SEC) on Superdex 200 10/300GL column, with PBS as running buffer. One addition of the linker was observed by Mass Spectrum. LCMS calculated: 58929; observed: [M + 1] 58929.

### Characterization of derivatized CRM_197_

Protein content was estimated by micro BCA (using BSA as standard and following manufacturer’s instructions [Thermo Scientifics]). HPLC-SEC analysis was used to compare derivatized protein with underivatized CRM_197_. All samples were eluted on TSK gel G3000 PWXL column (30 cm x 7.8 mm; particle size 7 µm; cod. 808021) with TSK gel PWXL guard column (4.0 cm x 6.0 mm; particle size 12 µm; cod.808033) (Tosoh Bioscience). The mobile phase was 0.1 M NaCl, 0.1 M NaH_2_PO_4_, 5% CH_3_CN, pH 7.2 at the flow rate of 0.5 mL/min (isocratic method for 30 min). Void and bed volume calibration was performed with λ-DNA (λ-DNA Molecular Weight Marker III 0.12–21.2 Kbp, Roche) and sodium azide (NaN_3_, Merck), respectively. Protein peaks were detected at 214 nm and 280 nm (UV detection) and using tryptophan fluorescence (emission spectrum at 336 nm, with excitation wavelength at 280 nm). Linker average loading on CRM_197_ was determined by MALDI-TOF analysis. For MALDI-TOF analysis, the protein was diafiltrated, using a Centricon-10 (Millipore), against NaH_2_PO_4_ 10 mM pH 7.2. Two microliters of protein (at a concentration of 5 mg/mL) were mixed with 2 µL of a saturated solution of sinapinic acid in 50% acetonitrile solution containing 0.1% TFA. Two microliters of the mix were spotted on a MTP 384 stainless steel target (Bruker Daltonics GmbH, Bremen, Germany) and allowed to air-dry. Measurements were recorded on an Ultraflex III (Bruker GmBH) MALDI-TOF/TOF MS in linear mode. External calibration was performed by spotting 2 µL of protein calibration standard II (Bruker Daltonics) containing the following proteins: trypsinogen (23,982 Da), protein A (44,613 Da) and bovine serum albumin (66,431 Da). All mass spectra were recorded by summing up to 400 laser shots. The Flex Analysis software packages provided by the manufacturer were used for data processing.

### Synthesis of OAg-cystamine-CRM_197_ conjugates

40 mg of 2192 OAg were solubilized in NaH_2_PO_4_ 100 mM pH 7.0 (40 mg/mL) and then cystamine (110 mg/mL, cystamine/OAg (w/w) = 2.75) and NaBH_3_CN (61 mg/mL, NaBH_3_CN/OAg (w/w) = 1.53) were added. The mixture was stirred for 3 h at RT and then desalted against water on a G-25 53 mL column. Cystamine disulfide bond was reduced by mixing the OAg at a concentration of 20 mg/mL with DTT 100 mM in NaH_2_PO_4_ 100 mM EDTA 5 mM for 1 h at RT. The derivatized OAg was purified by desalting on a G-25 53 mL column against 10 mM NaH_2_PO_4_ 5 mM EDTA pH 7.5. The amination reaction with cystamine was also performed at higher scale on 100 mg of OAg and the mixture purified by tangential flow filtration (10 k 200 cm^2^ Hydrosart membrane, 10 diafiltration cycles vs. NaCl 1M, followed by 10 cycles against water).

Derivatized OAg intermediates were characterized by phenol sulfuric assay for sugar content [27] and by HPLC-SEC [23] for verifying absence of aggregation or degradation after modification. After the reaction with cystamine, the introduction of NH_2_ groups was verified by TNBS colorimetric method [25] using 6-aminohexanoic acid as standard and subtracting the number of NH_2_ groups already present on the un-derivatized OAg sample. Percentage of OAg chains activated was calculated as moles of linked cystamine/moles of KDO (calculated by HPLC-SEC/semicarbazide assay [22]) %. After the reduction with DTT, introduction of SH groups was verified by Ellman analysis [28]. Activation on the terminus KDO was calculated as moles of linked SH/moles of KDO %, while the ratio % between SH groups/cystamine moles gave the efficiency of reduction reaction with DTT.

Conjugation was performed solubilizing the derivatized OAg in NaH_2_PO_4_ 100 mM EDTA 1 mM pH 7.2 (10 mg/mL), using a molar ratio of thiol groups to CRM_197_ of 30 to 1. After mixing ON at RT, the mixture was purified by size exclusion chromatography with a Sephacryl S-300 h column 1.6 cm x 90 cm [GE Healthcare], eluting with PBS pH 7.2 at 0.5 mL/min.

### Synthesis of OAg-propargylamine-CRM_197_

OAg (40 mg) was solubilized in NaH_2_PO_4_ pH 7.0 (40 mg/mL), then propargylamine (27.5 mg/mL, propargylamine/OAg (w/w) = 0.67) and NaBH_3_CN (62.8 mg/mL, NaBH_3_CN/OAg (w/w) = 1.53) were added. The reaction was stirred for 3 h at RT and purified by desalting on a G-25 53 mL column against water. Derivatized OAg intermediates were characterized by phenol sulfuric assay for sugar content [27] and by HPLC-SEC [23] for verifying absence of aggregation or degradation after modification.

Conjugation was performed adding by OAg-propargylamine to CRM_197_-N_3_ (5 mg/mL, average of 5.2 Lysines activated) in NaH_2_PO_4_ 400 mM pH 7.2 with THPTA 25 mM, sodium ascorbate 10 mM and CuSO_4_·5H_2_O 5 mM, with a molar ratio alkyne/azide = 5. Reaction was stirred at RT for 6 h and conjugate formation followed by HPLC-SEC (TSK gel 6000PW + TSK gel 5000PW).

### Synthesis of OAg-ADH-BCNesterI-CRM_197_

OAg (40 mg) was solubilized in AcONa 100 mM pH 4.5 (40 mg/mL), then ADH (48 mg/mL, ADH/OAg (w/w) = 1.2) and NaBH_3_CN (48 mg/mL, NaBH_3_CN /OAg (w/w) = 1.2) were added. The mixture was stirred for 2 h at 30 °C and then purified by desalting on a G-25 53 mL column against water.

For introduction of the second linker, BCN NHS ester I, OAg-ADH was dissolved in water/DMSO 1:9 (v/v) at a concentration of 50 mg/mL. When the derivatized OAg was completely solubilized, TEA was added (molar ratio TEA/total NH_2_ groups = 5; total NH_2_ groups included both phosphoethanolamine groups on the OAg and the hydrazide groups introduced with the linker ADH) followed by Click easy BCN NHS ester I (molar ratio BCN NHS ester I/total NH_2_ groups = 12). The solution was mixed at RT for 3 h. The sample was purified by desalting on a G-25 53 mL column against water.

Derivatized OAg intermediates were characterized by phenol sulfuric assay for sugar content [27] and by HPLC-SEC [23] for verifying absence of aggregation or degradation after modification. Introduction of NH_2_ groups was verified by TNBS colorimetric method [24, 25] using ADH as standard and subtracting the number of NH_2_ groups already present on the underivatized OAg sample. Free ADH was detected by RP-HPLC [29]. The percentage of OAg chains activated was calculated as moles of linked ADH (NH_2_)/moles of KDO % [22].

Total alkyne groups introduced with BCN NHS I were quantified by TNBS considering the residual number of unreacted NH_2_ groups remained after this derivatization step. Percentage of derivatization with BCN NHS I was calculated as molar ratio percentage of linked alkyne groups/total NH_2_ groups by TNBS before derivatization, indicating the percentage of moles of NH_2_ groups activated with this reaction.

For conjugation, CRM_197_-N_3_ at a concentration of 10 mg/mL in PBS was added of OAg-ADH-BCNesterI (molar ratio alkyne/azide = 4). Mixture was stirred for 6 h at RT. Conjugate purification was performed by hydrophobic interaction chromatography on a Phenyl HP column [GE Healthcare], loading 500 µg of protein for mL of resin in 50 mM NaH_2_PO_4_ 3M NaCl pH 7.2. The purified conjugate was eluted in water and the collected fractions were dialysed against 10 mM NaH_2_PO_4_ pH 7.2.

### OAg-CRM_197_ conjugates characterization

Total saccharide was quantified by phenol sulfuric assay [27], protein content by micro BCA and the ratio of saccharide to protein calculated. OAg-CRM_197_ conjugates profiles were compared with free CRM_197_ and free OAg by HPLC-SEC. All samples were eluted on a TSK gel 6000PW (30 cm x 7.5 mm) column (particle size 17 µm; Sigma 8-05765) connected in series with a TSK gel 5000PW (30 cm x 7.5 mm) column (particle size 17 µm; Sigma 8-05764) with TSK gel PWH guard column (7.5 mm ID x 7.5 cm L; particle size 13 µm; Sigma 8-06732) (Tosoh Bioscience). The use of the two columns in series gave better separation of conjugate from free saccharide and protein, allowing the conjugate to enter into the column. The mobile phase was 0.1 M NaCl, 0.1 M NaH_2_PO_4_, 5% CH_3_CN, pH 7.2 at the flow rate of 0.5 mL/min (isocratic method for 60 min). OAg peaks were detected by dRI, while UV detection at 214 nm and 280 nm was used for free protein and conjugate detection. Protein and conjugate peaks were also detected using tryptophan fluorescence (emission spectrum at 336 nm, with excitation wavelength at 280 nm). Free protein was estimated by HPLC-SEC, running a calibration curve of the unconjugated protein in the range 5–50 µg/mL under the same conditions as for the conjugate. The percentage of unconjugated CRM_197_ was calculated by dividing the amount of free protein detected by HPLC-SEC by the total amount of protein quantified in the sample by micro BCA. Free saccharide was verified to be lower than 20% by comparing eventual peak of free OAg in the conjugate with the peak of an OAg standard injected at the concentration corresponding to 20% of the total sugar amount as estimated by phenol sulfuric assay.

## Results

### Thiol conjugation chemistry

**Fig. 1:**
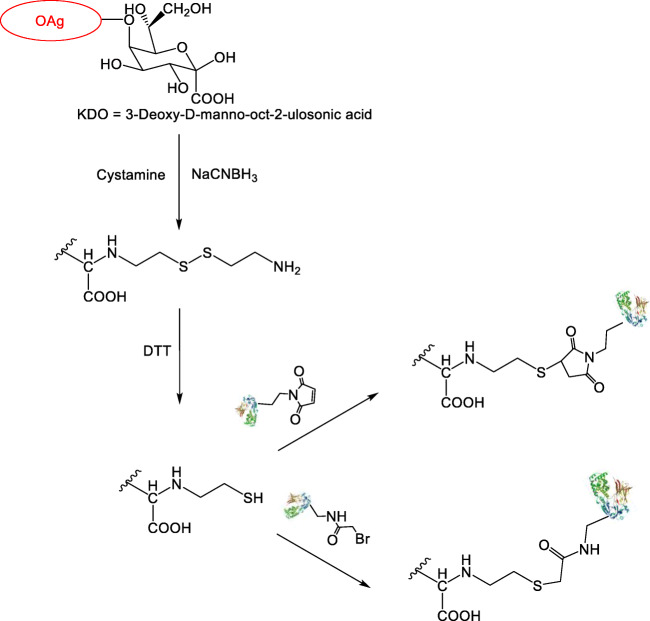
Thiol conjugation chemistry. Activation of the terminal KDO unit of the OAg chain with cysteamine and conjugation to activated-CRM_197_

Thiol-conjugation chemistry was initially tested. The KDO moiety was targeted for introducing cystamine linker at the reducing end of the OAg chain without modifying the repeating units structure. After DTT-reduction, the activated OAg was conjugated to the carrier protein (Fig. 1).

OAg derivatization with cystamine was characterized by sugar recovery higher than 80%. TNBS analysis indicated that 70–80% of OAg chains were activated. After DTT addition, sugar recovery was again higher than 75% and a complete reduction of the thiols was confirmed by the analysis of thiol groups introduced.

**Fig. 2:**
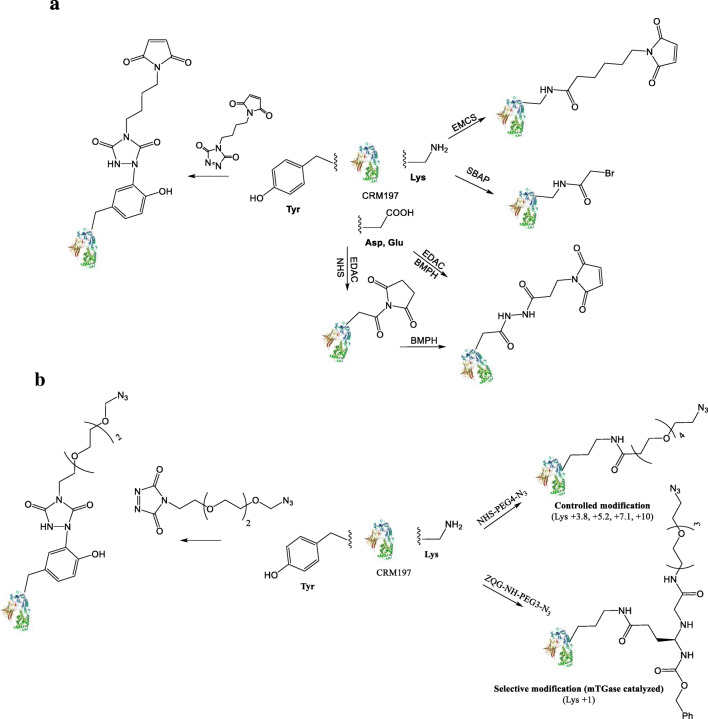
Activation of the carrier protein. (**A**) Thiol chemistry: CRM_197_ was derivatized on Lys, Tyr or Glu/Asp using different hetero bi-functional linkers able to react with cysteamine-activated OAg. (**B**) Click Chemistry: CRM_197_ was derivatized on Lys or Tyr with NHS-PEG4-N_3_ to introduce azido groups. Lysine activation was performed by selective (Lys + 1) or controlled modification (Lys)

CRM_197_ was derivatized using different hetero bi-functional linkers, with a functional group able to react with the thiol unit introduced at the end of the OAg chain. Different amino acids were targeted, also trying to result in variable protein loading (Fig. 2A; Table 1). In all cases, protein recovery was higher than 90%. Overall, derivatization on Lysine residues allow to introduce the highest number of linkers per mole of protein, respectively 8 using SBAP and 11.3 using EMCS (1–2, Table 1). Using controlled conjugation chemistries, an average of 3.1 lysines were instead targeted with EMCS (3, Table 1). Reaction of CRM_197_ with BMPH by EDAC chemistry resulted in the introduction of an average number of 3.4 linkers per protein (4, Table 1). When NHS was added to EDAC for activating the COOH groups on the protein, trying to increase the activation degree, an average of 5.1 linkers was introduced (5, Table 1). Tyrosines were also modified obtaining an average loading of 4.1 linkers per protein (6, Table 1).

**Table 1:** OAg-CRM197 conjugation by thiol chemistry

Conjugate	Chemistry	Target	Average CRM_197_ labeling	OAg chains per CRM_197_	Conjugate (%)
1 - OAg-cysteamine-SBAP-CRM_197_	Thiol (Alkylation)	Lys	+ 8.0	1.6	100
2 - OAg-cysteamine-EMCS-CRM_197_	Thiol (Addition)	Lys	+ 11.3	2.7	100
3 - OAg-cysteamine-EMCS-CRM_197_	Thiol (Addition)	Lys	+ 3.1	-	No
4 - OAg-cysteamine-BMPH-CRM_197_	Thiol (Addition)	Glu/Asp	+ 3.4	-	No
5 - OAg-cysteamine-(NHS)BMPH-CRM_197_	Thiol (Addition)	Glu/Asp	+ 5.1	-	No
6 - OAg-cysteamine-maleimide-CRM_197_	Thiol (Addition)	Tyr	+ 4.1	-	No

Thiolated OAg was then conjugated to derivatized CRM_197_ characterized by different amino acids activated (Lys, Tyr or Glu/Asp) and different linker loading. Using this conjugation chemistry, it was not possible to obtain conjugate formation unless the number of linkers per protein was at least 8 (1–2, Table 1; Fig. 1).

The two conjugation reactions that succeeded, both based on lysine chemistry, were characterized by no presence of unreacted protein in the conjugation mixture. Even if the number of linkers per CRM_197_ was high (8 and 11.3), corresponding conjugates were characterized by a low OAg to protein molar ratio (1.6 and 2.7 respectively) (1–2, Table 1).

Looking at the HPLC-SEC profiles (dRI) of the conjugation mixtures, even when conjugation was successful, the presence of a sugar population at higher molecular weight was observed, probably deriving from oxidation of the thiolated OAg to generate a sugar dimer (not shown). Different methods were attempted to avoid the generation of the oxidation product, and hence of an unreactive OAg form, to see if this could then result in higher conjugation efficiency. Conjugation of DTT-treated OAg-cystamine with CRM_197_-BMPH under N_2_ caused precipitation of the protein; one-pot-two-step OAg-cystamine reduction with TCEP as reducing agent and conjugation to CRM_197_-BMPH (5.2 linkers) was also not successful. When DTT-treated cystamine was conjugated with CRM_197_-BMPH in the presence of TCEP, the formation of the disulfide aggregate was avoided, however most of protein did not conjugate.

In summary thiol conjugation strategies did not allow to get all the panel of wanted conjugates and it became needed to identify a different conjugation approach.

### Click chemistry

There are two main ways of performing the click 1,3-dipolar reaction: by using metal as a catalyst (usually copper), or alternatively lowering the activation barrier for [3 + 2] cycloaddition by employing intrinsically highly strained cyclic alkynes that readily react with azide groups. For starting, both the approaches were compared for the reaction of the alkyne-derivatized *S*. Typhimurium OAg with CRM-N_3_.

**Fig. 3:**
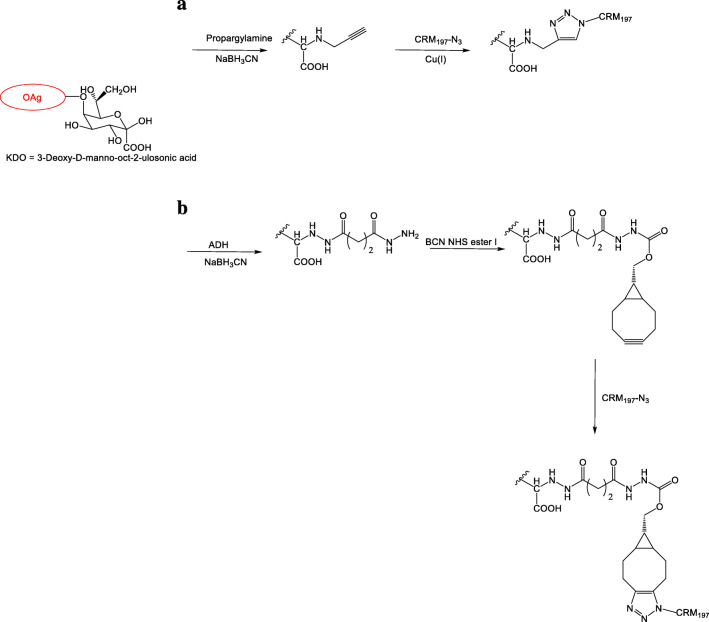
Click conjugation chemistry. Two conjugation strategies: (**A**) Copper-free click reaction. (**B**) Copper-catalyzed click reaction

For the copper click chemistry, the OAg was derivatized with propargylamine by reductive amination on the terminal KDO (Fig. 3A). For the copper-free approach instead, the alkyne linker was introduced on the OAg-ADH, with activation higher 80% (Fig. 3B).

The inclusion of azido groups on CRM_197_ was initially performed by targeting lysines using NHS-PEG4-N_3_ (Fig. 2B, controlled modification). Different loadings were obtained, in the range of 3.8–10 linker introduced per protein, depending on the amount of linker added. In all cases, protein recovery was higher than 90% (Table 2). CRM_197_ with an average number of 5.2 linkers introduced (CRM_197_-N_3_(LYS5.2)) was used at 5 mg/mL for comparing these two approaches.

**Table 2:** CRM197 controlled modification by azide insertion on lysines

Linker:lysine ratio (mol/mol)	Theoretical Lys labelling*	Average Lys labelling
0.14	+ 5.5	+ 3.8
0.18	+ 7.0	+ 5.2
0.26	+ 10.1	+ 7.1
0.39	+ 15.2	+ 10

*Calculated considering 39 lysines on CRM_197_

Despite a lower sugar to protein ratio, the copper free conjugation was more efficient (Table 3). In both cases, reaction time did not seem to have a strong impact on conjugate formation (Table 3). Because of the higher conjugation efficiency and to avoid the use of a toxic metal, the copper-free approach was selected for further experiments.

**Table 3:** Reaction conditions used for the comparison of copper-free and copper-catalyzed conjugation

Copper	Alkyne:azide ratio (mol/mol)	Reaction time (h)	Conjugate (%)
Yes	5	2	28
		4	36
		6	40
No	1	2	69
		4	75
		6	78

CRM_197_-N_3_(LYS5.2) at 5 mg/ml

**Fig. 4:**
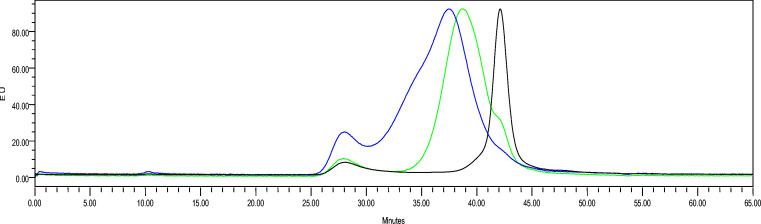
HPLC-SEC profiles of OAg-ADH-BCNesterI-CRM_197_ conjugation with CRM_197_-N_3_(LYS5.2), green, or CRM_197_-N_3_(LYS10), blue, in comparison to free CRM_197_, black. Conjugation mixture were analyzed after 6 h at RT, using carrier protein at 10 mg/mL and alkyne/azide molar ratio of 4

Maintaining protein concentration at 5 mg/mL and increasing the alkyne to azide molar ratio from 1 to 2, conjugation efficiency was not altered after 6 h of reaction, with 78% of CRM_197_ conjugated (Table 4). Increasing protein concentration from 5 to 10 mg/mL and the alkyne to azide molar ratio from 2 to 4, conjugation became quantitative. When a protein with a higher average number of linkers was used (from 5.2 to 10), no free CRM_197_ was detected in the conjugation mixture (Table 4; Fig. 4).

**Table 4:** Reaction conditions optimized for the copper-free conjugation

Azide per CRM_197_	[CRM_197_] (mg/mL)	Alkyne:azide (mol/mol)	Conjugate (%)
5.2	5	1:1	78
5.2	5	2:1	78
5.2	10	4:1	90
10	10	4:1	100

Reaction time is 6 h

The optimal conditions identified here for click-chemistry conjugation were applied for the synthesis of a panel of glycoconjugates differing for type of amino acid targeted and number of sites activated on the protein (Table 5; Fig. 2B) [6]. In order to compare the conjugates generated by the free-metal click and the thiol conjugation chemistry with a similar linker loading on the carrier protein, CRM_197_ with an average number of 7.1 linkers introduced (CRM_197_-N_3_(LYS7.1)) was considered (7, Table 5). As previously reported, the conjugation to the alkyne-derivatized OAg was successful with all CRM_197_ conjugated and residual free OAg removed by purification through HIC (Table 5) [6]. The conjugate was characterized by a similar OAg to protein molar ratio (2.0) to what previously observed with thiol chemistry on activated lysine groups on CRM_197_. We have determined that click chemistry can be used for the synthesis of conjugate vaccines when the linker loading is low targeting both lysines and tyrosine that were immunogenic in mice (8–10 Table 5; Fig. 2B) [6]. Importantly, the reaction conditions allowed the conjugation with only one linker CRM_197_-N_3_(LYS1), where 38% of CRM_197_ was conjugated after 6 h using a chemoenzymatic approach (8 Table 5; Fig. 2B selective modification).

**Table 5:** OAg-CRM197 conjugation by click chemistry

Conjugate	Chemistry	Target	Average CRM_197_ labeling	OAg chains per CRM_197_	Conjugate (%)
7 - OAg-ADH-BCNesterI-CRM_197_	Click (Copper-free)	Lys	+ 7.1	2.0	100
8 - OAg-ADH-BCNesterI-CRM_197_	Click (Copper-free)	Lys	+ 1	0.7	31
9 - OAg-ADH-BCNesterI-CRM_197_	Click (Copper-free)	Lys	+ 3.8	1.5	91
10 - OAg-ADH-BCNesterI-CRM_197_	Click (Copper-free)	Tyr	+ 4.3	3.7	81
*Data from [6]					

As previously observed with CRM_197_-N_3_(LYS5.2), the conjugation of CRM_197_-N_3_(LYS1) is fast as increasing the reaction time from 2 to 6 h did not impact the conjugate formation (Table 6).

**Table 6:** Effect of reaction time on copper-free click conjugation with CRM197-N3(LYS1)

Reaction time (h)	Conjugate (%)
2	34
4	35
6	38
CRM_197_-N_3_(LYS1) is at 10 mg/mL, alkyne:azide is 4:1 (mol/mol)	

## Discussion

The design of structurally defined glycoconjugates brings advantages in terms of physicochemical characterization of the vaccine and could lead to the selection of candidates with enhanced efficacy. With this regard, we have recently demonstrated that the conjugation site plays a role in determining the immunogenicity in mice and one single attachment point may be sufficient to induce high levels of bactericidal antibodies [6]. The use of structurally defined constructs with epitopes displayed at precise sites is also desirable to better understand the antigen presentation to the immune system and to rationalize the development of efficacious glycoconjugates vaccines. However, selective chemistries are usually characterized by lower conjugation efficiency than random ones.

The possibility of applying click chemistry [30] in bioconjugation was first demonstrated for the preparation of peptidotriazoles via solid phase synthesis [31]. Recently, it has been applied to the synthesis of glycoconjugate vaccines [5, 6, 18, 32]. One of these reactions, the Huisgen 1,3-dipolar cycloaddition [33], has become the most popular click reaction, especially because it can proceed rapidly at room temperature by employing copper as a catalyst [31, 34]. It is characterized by mild reaction conditions, high yields and simple work-up, selectivity, specificity and can be usually performed in water. One of the most important properties of the click chemistry is its bio-orthogonality. The azide moiety is absent in almost all naturally existing compounds, lacks reactivity with natural biomolecules and undergoes ligation only with a limited set of functionalities, such as alkyne groups. The sulfhydryl group is a popular target in many modification strategies. The frequency of sulfhydryl occurrence in proteins or other molecules is usually very low compared to other groups like amines or carboxylates. Therefore, the use of sulfhydryl-reactive chemistries can also restrict modification to only a limited number of sites within a target molecule.

In this study we have screened different click and thiol conjugation chemistries with the aim to identify a powerful conjugation methodology for the synthesis of site-selective glycoconjugates targeting one single point of the saccharide chain and one-to-few points of attachment on the carrier protein. We have used *S.* Typhimurium OAg and CRM_197_ as carbohydrate hapten and carrier protein models. It is worth mentioning that *S.* Typhimurium OAg and CRM_197_ are large molecules (20.5 kDa and 58.4 kDa, respectively), therefore the possibility to design site-selective constructs is hardly challenged by steric hindrance factors. Click chemistry has proved to be a more powerful tool in this sense, allowing the synthesis of conjugates even where only one position on the protein was available for linkage to the KDO unit of the sugar chain. When using thiol-chemistry, we were not successful in obtaining glycoconjugates unless the number or linkers on the protein was at least 8. Possible reasons to explain this behavior could be related with the instability of the functional groups on the linker in the presence of CRM_197_ and the verified oxidation of thiolated OAg in the conjugation conditions.

Indeed, insertion of thiol groups in meningococcal C oligomers followed by thiol-maleimide conjugation on CRM_197_ has been previously shown feasible, although the conjugation with CRM_197_-EMCS (6.5 linker per protein) reaction linked on average only 1.8 oligosaccharides per protein. In our work we have confirmed that conjugation of *S*. Typhimurium OAg to CRM_197_-EMCS is feasible, provided that a high number of linkers per protein are present (+ 11.3), resulting in a conjugate bearing about 2.7 OAg chain per protein. We did not observe conjugation in the presence of only 3.1 linker per protein. While in our investigation we did not test CRM_197_-EMCS with an average of 6.5 linkers and we have used a longer sugar model (20.5 vs. 4.5 kDa), we think that both finding underline how, in the presence of thiol chemistry, the synthesis of conjugate vaccine with a controlled number of glycopeptide linkages may present some issues [35]. In addition, work from Nilo et al. confirmed that thiol-maleimide chemistry resulted in lower sugar loading on CRM_197_ compared to copper-free conjugation [26].

Copper-free chemistry was preferred because it produced better conjugation yields in preliminary tests and avoids the use of a toxic metal. Differently from the thiol chemistry tested, copper-free click chemistry allowed the linkage of the terminal sugar end of the OAg chain to the protein with high conjugation yields (higher than 80%) even when few linkers were present on the protein (average number of 4) and to have conjugate formation with just one linker on the protein (38% of CRM_197_ conjugated). It is important also to consider that the conditions tested for the thiol chemistry have an intrinsic bias, since they used higher concentration of both OAg and protein, and higher molar ratio of active groups of OAg per linker on the carrier protein. The click conjugation proceeds with a fast rate with the reaction completed in only 2 h even when only one linker is present on the carrier protein. However, the linkers used for the synthesis of click and thiol conjugates were different, and an impact of the type of linker on conjugate formation cannot be excluded. While it has been previously reported that the linkers used for thiol-maleimide addition induced a lower anti-linker response compared to the cyclooctene ring generated by SPAAC [26], our group has previously shown how conjugate with even only one sugar moiety per protein synthesized by copper-free click reaction can induce strong immunogenicity [6].

In order to generalize the outcome of the comparison between thiol and click chemistries, different sugar and – more importantly - protein models should be investigated. While we would expect different carrier proteins to behave differently, we think that the outcome of the current investigation will likely apply to the diphtheria toxoid (DT), another commonly used carrier protein, structurally related to CRM_197_.

In conclusion, we compared thiol and click chemistry for the synthesis of site-selective conjugates on the OAg. Click chemistry allows the synthesis of glycoconjugates where different amino acid can be targeted and in a different number, impacting the number of sugar present per carrier protein and the immunogenicity [6]. Click chemistry is therefore a powerful tool for the synthesis of glycoconjugate vaccines as it allows the investigation of different parameters important for the immunogenicity.
